# Effects of Empagliflozin and Dapagliflozin on Lipid Profiles and Atherogenic Risk Indices in Patients with Heart Failure and a History of CABG: First Evidence in the Literature

**DOI:** 10.3390/jcm14207395

**Published:** 2025-10-20

**Authors:** Ilhan Ozgol, Ece Yigit Gencer, Cennet Yildiz, Dilay Karabulut, Fatma Nihan Turhan Çaglar, Burcu Bicakhan, Cihan Yucel, Serkan Ketenciler, Asime Ay, Zerrin Yigit

**Affiliations:** 1 Department of Cardiovascular Surgery, Prof. Dr. Cemil Tascıoglu City Hospital, 34348 Istanbul, Turkey; 2 Department of Internal Medicine, Pendik Medipol University Hospital, Faculty of Medicine, Istanbul Medipol University, 34893 Istanbul, Turkey; 3 Department of Cardiology, Bakırkoy Dr. Sadi Konuk Training and Research Hospital, 34147 Istanbul, Turkey; 4 Department of Cardiovascular Surgery, Gaziosmanpasa Training and Research Hospital, 34255 Istanbul, Turkey; 5 Department of Anesthesia and Reanimation, Prof. Dr. Cemil Tascıoglu City Hospital, 34348 Istanbul, Turkey; 6 Department of Cardiology, Faculty of Medicine, Istanbul University Cerrahpasa, 34098 Istanbul, Turkey

**Keywords:** empagliflozin, dapagliflozin, SGLT2 inhibitors, heart failure, coronary artery bypass grafting (CABG), lipid profile, atherogenic risk indices

## Abstract

**Objective:** This study aimed to compare the effects of empagliflozin and dapagliflozin on classical lipid parameters—including total cholesterol (TC), low-density lipoprotein cholesterol (LDL-C), high-density lipoprotein cholesterol (HDL-C), and triglycerides (TG)—as well as on atherogenic risk indices, including the atherogenic index of plasma (AIP), Castelli Risk Index I (CRI-I), Castelli Risk Index II (CRI-II), atherogenic coefficient (AC), and triglyceride-glucose index (TyG), in patients with heart failure and a history of coronary artery bypass grafting (CABG). To our knowledge, this is the first study to comprehensively evaluate these parameters in this high-risk population. **Methods:** This single-center, retrospective study included 484 patients with preserved ejection fraction heart failure and prior CABG who were treated with sodium–glucose cotransporter-2 (SGLT2) inhibitors. Patients were allocated to empagliflozin (*n* = 201) or dapagliflozin (*n* = 283) groups. All patients were receiving statin therapy. Lipid parameters and atherogenic indices were evaluated at baseline and after 12 weeks of treatment. **Results:** Both empagliflozin and dapagliflozin significantly reduced TC and LDL-C at 12 weeks (*p* < 0.001). No significant changes were observed in HDL-C or TG. Both agents produced significant improvements in CRI-I, CRI-II, AC, and TyG index (all *p* < 0.001), while AIP remained unchanged. Dapagliflozin achieved a greater reduction in TC (*p* = 0.044). **Conclusions:** This study represents the first direct comparison of empagliflozin and dapagliflozin on lipid profiles and atherogenic indices in patients with heart failure and prior CABG. Both agents significantly improved TC, LDL-C, and atherogenic indices. Dapagliflozin achieved a greater reduction in TC compared with empagliflozin, but overall both drugs demonstrated favorable and largely comparable effects. Beyond improvements in absolute values, both agents also contributed to favorable shifts in risk categories of lipid-derived indices. These findings suggest that clinical decision-making between empagliflozin and dapagliflozin may rely on factors other than lipid modulation. Larger multicenter prospective trials are warranted to confirm these results and clarify their long-term cardiovascular implications.

## 1. Introduction

Sodium–glucose cotransporter 2 inhibitors (SGLT2i) selectively and reversibly inhibit SGLT2 in the proximal renal tubules, thereby reducing glucose reabsorption [1,2]. These agents lower plasma glucose independently of insulin, providing glycemic control without hypoglycemia and exerting additional cardiovascular benefits. This provides glycemic control without increasing the risk of hypoglycaemia and also exerts beneficial effects on cardiovascular morbidity and mortality [1,2,3,4,5,6,7,8,9].

Beyond their glucose-lowering capacity, SGLT2 inhibitors have recently been suggested to modulate lipid metabolism through mechanisms independent of glycemia, potentially improving or preventing dyslipidemia [10,11]. Proposed mechanisms include regulation of lipid synthesis and transport, reduction in tissue lipid accumulation and oxidative stress, improvement in insulin sensitivity, a metabolic shift from carbohydrate to lipid utilization with increased ketone production, decreased hepatic lipogenesis, and reduced intestinal cholesterol absorption [3,11,12,13,14,15,16,17].

However, the literature presents heterogeneous findings regarding the impact of SGLT2i on lipid profiles, particularly low-density lipoprotein cholesterol (LDL-C). While some studies have reported increases in LDL-C, others have described overall cardiovascular benefits attributed to complex regulatory mechanisms such as modulation of lipoprotein subfractions [18,19,20,21]. Thus, consensus has not yet been reached on the net effect of SGLT2 inhibitors on the initiation and progression of atherosclerosis.

Cardiovascular disease remains a leading cause of morbidity and mortality worldwide, with atherosclerosis and dyslipidemia as key contributors. In this context, not only classical lipid parameters but also indices derived from these measures have gained prominence in cardiovascular risk assessment. The atherogenic index of plasma (AIP), calculated as log(TG/HDL-C), has been identified as a reliable predictor of coronary artery disease risk beyond traditional lipid levels [22,23,24]. This index is considered a surrogate marker of small, dense LDL-C particles and reflects the balance between atherogenic and protective lipoproteins [25,26].

Other widely used indices include the Castelli Risk Index I (CRI-I, TC/HDL-C), which reflects atherosclerotic plaque burden [27], and Castelli Risk Index II (CRI-II, LDL-C/HDL-C), another strong predictor of cardiovascular risk [28]. Both indices are independent risk factors for coronary artery disease and predictive of future cardiovascular events. The Atherogenic Coefficient (AC, non-HDL-C/HDL-C) is also recognized as an independent predictor of coronary artery disease [29], while non-HDL cholesterol (non-HDL-C) serves as a surrogate of apolipoprotein B levels and correlates more strongly with cardiovascular risk than LDL-C alone [30]. The triglyceride–glucose (TyG) index, calculated as ln(TG × fasting glucose/2), has been validated as a simple marker of insulin resistance and a predictor of coronary artery calcification [31,32,33,34].

Given these considerations, and in light of the potential clinical relevance of lipid-derived indices, we aimed in this retrospective study to compare the effects of empagliflozin and dapagliflozin on classical lipid parameters (total cholesterol [TC], LDL-C, high-density lipoprotein cholesterol [HDL-C], triglycerides [TG]) and atherogenic indices (AIP, CRI-I, CRI-II, AC, TyG) in patients with heart failure and a history of coronary artery bypass grafting (CABG). By doing so, we sought to contribute to a better understanding of the differential effects of SGLT2 inhibitors on dyslipidemia and cardiovascular risk markers.

## 2. Materials and Methods

**Figure 1:**
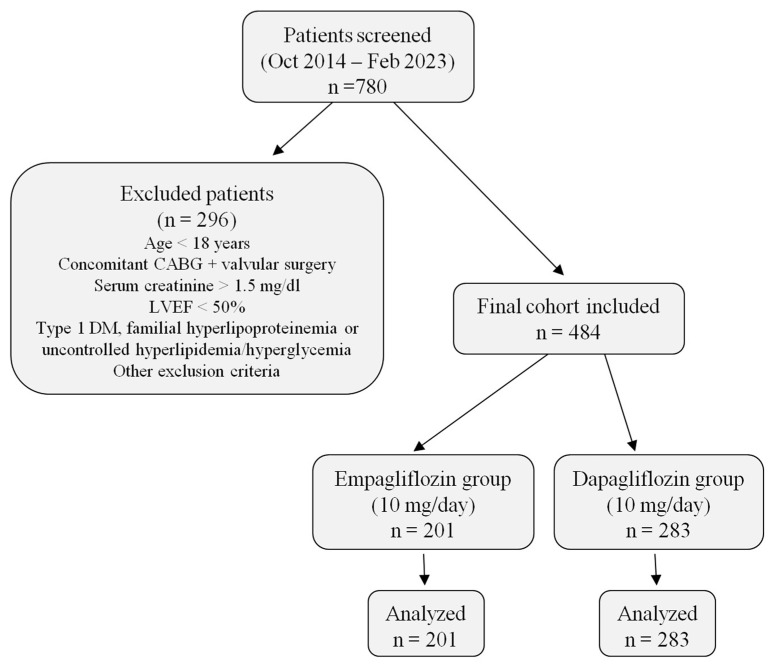
Patient flow diagram. The figure summarizes patient screening, exclusion criteria, and final allocation into empagliflozin and dapagliflozin groups, including the number of patients analyzed in each arm. Abbreviations: CABG, coronary artery bypass graft; LVEF, left ventricular ejection fraction; DM, diabetes mellitus.

In this single-center, retrospective study, a total of 780 patients with heart failure who were followed between October 2014 and February 2023 and treated with SGLT2i were screened for eligibility. Of these, 296 patients were excluded according to predefined criteria. The final study cohort comprised 484 patients with preserved ejection fraction (LVEF ≥ 50%), all of whom required additional therapy for heart failure despite ongoing treatment. Among them, 201 received empagliflozin (10 mg/day) and 283 received dapagliflozin (10 mg/day). All patients were followed for 12 weeks, and complete baseline and follow-up data were available for analysis (Figure 1).

Exclusion criteria included: age < 18 years; type 1 diabetes mellitus; previous valvular surgery; serum creatinine >1.5 mg/dL; chronic hepatitis, malignancy, chronic organ failure, rheumatologic disease, vasculitis, splenomegaly, hypersplenism, alcohol or substance abuse, corticosteroid use, familial hyperlipoproteinemia, uncontrolled hypertension, hyperlipidemia or hyperglycemia; ketoacidosis or diabetic coma within 6 months prior to therapy; and use of agents affecting lipid profiles (niacin, thyroid hormone, omega-3 ethyl esters, vitamins D or E) within the previous 3 months. Patients with triglyceride levels ≥500 mg/dL were excluded due to the unreliability of LDL-C calculation by the Friedewald formula.

Blood samples were collected after 10–12 h of fasting and analyzed at the hospital’s central laboratory. LDL-C was calculated using the Friedewald formula. Echocardiographic examinations were performed in the left lateral decubitus position according to the American Society of Echocardiography guidelines, using a Vivid E9 echocardiography system (GE Healthcare, Chicago, IL, USA) the Simpson’s biplane method from apical two- and four-chamber views. Preserved ejection fraction was defined as LVEF ≥ 50%. The diagnosis of heart failure was based on clinical signs and symptoms supported by structural/functional abnormalities, including diastolic dysfunction or elevated filling pressures, as well as natriuretic peptide levels.

Collected variables included: TC, LDL-C, HDL-C, TG, fasting glucose, HbA1c, uric acid, creatinine, demographic data (age, sex, smoking), comorbidities (hypertension, diabetes mellitus, hyperlipidemia), and concomitant medications. Atherogenic indices were manually calculated using the following formulas and thresholds:

- AIP: log_10_(TG/HDL-C); low risk < 0.11, intermediate risk 0.11–0.21, high risk ≥ 0.21.
- CRI-I: TC/HDL-C; low risk < 3.5.
- CRI-II: LDL-C/HDL-C; low risk < 3.0.
- AC: (TC–HDL-C)/HDL-C or non-HDL-C/HDL-C; low risk < 3.0.
- TyG index: ln(TG × fasting glucose/2); low risk < 4.5.

Heart failure therapy included angiotensin-converting enzyme inhibitors (ACEIs), angiotensin receptor blockers (ARBs), beta-blockers, diuretics, and mineralocorticoid receptor antagonists. All patients with coronary artery disease received acetylsalicylic acid and were on statin therapy for at least 3 months according to the American College of Cardiology/American Heart Association (ACC/AHA) guidelines. Patients who had a change in statin regimen within the previous month were excluded.

After 12 weeks of SGLT2i therapy, lipid parameters (TC, LDL-C, HDL-C, TG) and atherogenic indices (AIP, CRI-I, CRI-II, AC, TyG) were reassessed and compared with baseline values. Differences between empagliflozin and dapagliflozin were analyzed.

The study was conducted in accordance with the Declaration of Helsinki and approved by the Ethics Committee of Bakırköy Dr. Sadi Konuk Training and Research Hospital (Protocol No.: 2024/348, Decision No.: 2024-13-16).

### Statistical Analysis

All statistical analyses were performed using IBM SPSS Statistics for Windows, version 27.0 (IBM Corp., Armonk, NY, USA). Continuous variables were expressed as mean ± standard deviation (SD) or median with interquartile range (IQR), and categorical variables as counts and percentages. The Kolmogorov–Smirnov test was used to assess the normality of data distribution. For normally distributed data, comparisons between groups were made using Student’s *t*-test, whereas the Mann–Whitney U test was applied for non-normally distributed variables. Categorical variables were compared using the chi-square test or Fisher’s exact test, as appropriate. Within-group comparisons between baseline and week 12 were conducted using paired *t*-tests (for normally distributed variables) or Wilcoxon signed-rank tests (for non-normally distributed variables). Repeated-measures ANOVA was applied to evaluate changes over time and time–group interactions. ANCOVA models were performed to adjust for baseline imbalances in selected clinical variables, in order to verify the robustness of the findings. A two-tailed *p*-value < 0.05 was considered statistically significant.

## 3. Results

### 3.1. Patient Characteristics

A total of 484 patients with heart failure and a history of CABG were retrospectively included in the study (empagliflozin: *n* = 201; dapagliflozin: *n* = 283). There was no significant difference in mean age between the groups (EMPA: 61.4 ± 10.6 years; DAPA: 63.1 ± 11.2 years; *p* = 0.151), nor in the proportion of patients over 60 years of age (58.2% vs. 59.4%). The sex distribution was also similar (59.7% vs. 65.7%; *p* = 0.176).

There was no significant difference between groups in terms of comorbidities (hypertension, diabetes mellitus, hyperlipidaemia, smoking, and atrial fibrillation) or concomitant medications. All patients were receiving acetylsalicylic acid and statin therapy, with the majority on high-dose regimens and no significant difference between groups in the distribution of high- versus moderate-dose statin use (*p* = 0.409) (see Table 1).

### 3.2. Biochemical Parameters and Atherogenic Risk Indices

Baseline fasting blood glucose and HbA1c levels were comparable. In the dapagliflozin group, however, BUN (18.7 ± 8.3 mg/dL; *p* = 0.030), creatinine (0.97 ± 0.22 mg/dL; *p* = 0.001), and uric acid (6.35 ± 1.79 mg/dL vs. 5.74 ± 1.55 mg/dL; *p* = 0.001) levels were slightly higher.

No significant differences were observed between the groups in terms of TC (193.8 ± 50.3 vs. 186.2 ± 49.6 mg/dL; *p* = 0.087) or LDL-C (130.6 ± 45.6 vs. 123.3 ± 44.5 mg/dL; *p* = 0.061). However, HDL-C was lower in the dapagliflozin group (46.4 ± 15.4 vs. 43.6 ± 13.6 mg/dL; *p* = 0.018). TG levels were similar. The AIP, CRI-I, CRI-II, AC, and TyG indices showed no significant difference between the groups at baseline (see Table 1).

### 3.3. Changes in Lipid Parameters and Atherogenic Indices (12th Week)

Remarkable decreases in classical lipid parameters were observed in both treatment groups during the follow-up period. In the empagliflozin group, TC decreased from 193.8 ± 50.3 mg/dL at baseline to 169.7 ± 44.6 mg/dL at week 12. In the dapagliflozin group, it decreased from 186.2 ± 49.6 mg/dL to 162.3 ± 38.3 mg/dL. This reduction was statistically significant in both groups (*p* < 0.001 for both), with a mild but significant difference in favour of the dapagliflozin group at week 12 (*p* = 0.044).

**Figure 2:**
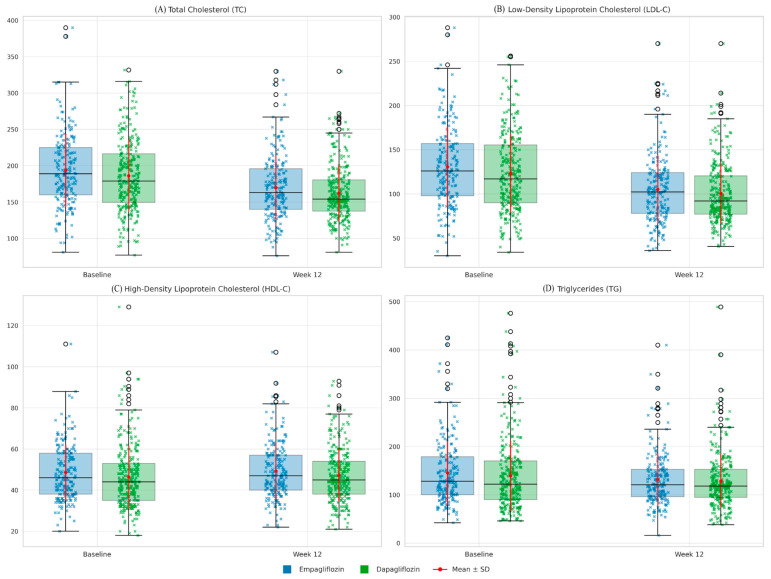
Lipid profiles (Baseline vs. Week 12) in patients receiving empagliflozin or dapagliflozin. Boxplots with overlaid strip plots and mean ± SD markers illustrate total cholesterol (TC), low-density lipoprotein cholesterol (LDL-C), high-density lipoprotein cholesterol (HDL-C), and triglycerides (TG) at baseline and after 12 weeks of treatment. Blue indicates empagliflozin, and green indicates dapagliflozin. Blue and green X denote individual values for empagliflozin and dapagliflozin, respectively. Red dots with error bars represent group means ± SD. Both empagliflozin and dapagliflozin significantly reduced TC and LDL-C, while no significant changes were observed in HDL-C or TG. No clinically meaningful differences were observed between the two drugs.

Similarly, LDL-C levels decreased from 130.6 ± 45.6 mg/dL to 105.2 ± 38.7 mg/dL in the empagliflozin group and from 123.3 ± 44.5 mg/dL to 100.4 ± 34.2 mg/dL in the dapagliflozin group (*p* < 0.001 for both). No significant difference was found between the two groups at the end of the treatment period (*p* = 0.132). HDL-C levels increased slightly in both groups compared with baseline, but this change was not statistically significant (*p* > 0.05). TG levels decreased by approximately 10–15 mg/dL in both groups, but these changes were not statistically significant either (*p* > 0.05). Changes in lipid profiles from baseline to week 12 are shown in Table 2 and Figure 2.

**Figure 3:**
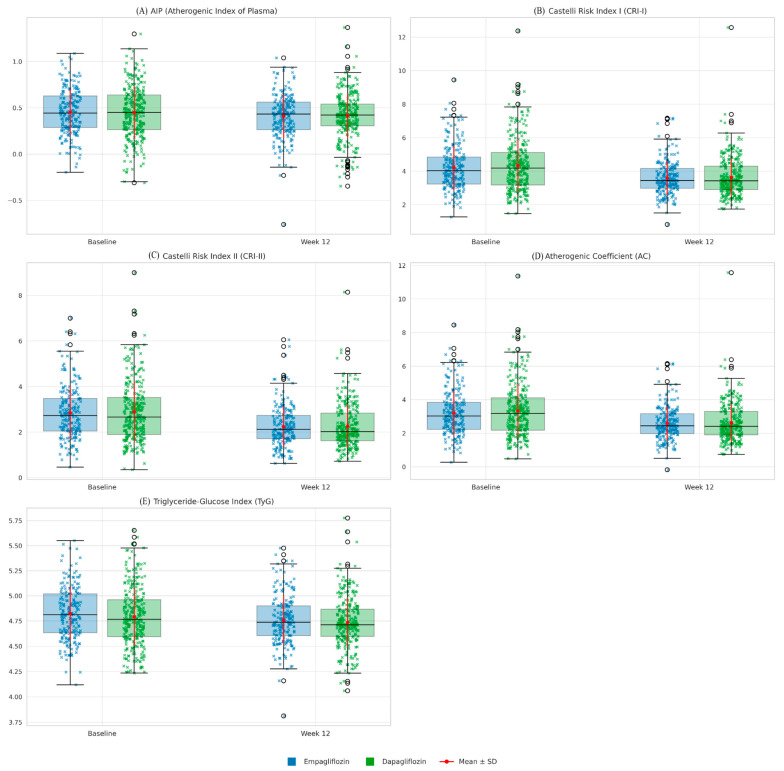
Comparative changes in atherogenic indices (Baseline vs. Week 12) in patients treated with empagliflozin or dapagliflozin. Boxplots with overlaid strip plots represent the distribution of individual values for each index at baseline and after 12 weeks of treatment, stratified by drug group (blue: empagliflozin, green: dapagliflozin). Blue and green X denote individual values for empagliflozin and dapagliflozin, respectively. Red dots with error bars indicate mean ± standard deviation. Both empagliflozin and dapagliflozin significantly reduced Castelli Risk Index I (CRI-I), Castelli Risk Index II (CRI-II), the atherogenic coefficient (AC), and the triglyceride-glucose index (TyG), whereas no significant change was observed in the atherogenic index of plasma (AIP). Improvements were comparable between the two drugs, with no statistically significant differences detected.

In terms of lipid-derived formulas, the AIP value was 0.45 ± 0.25 at baseline, decreasing slightly to 0.41 ± 0.24 by week 12. This improvement was not statistically significant (*p* > 0.05). By contrast, the CRI-I, CRI-II, AC, and TyG indices decreased significantly in both groups. In the empagliflozin group, these values decreased as follows: CRI-I from 4.21 ± 1.33 to 3.60 ± 1.01; CRI-II from 2.85 ± 1.16 to 2.24 ± 0.90; AC from 3.21 ± 1.33 to 2.60 ± 1.01; and TyG from 4.82 ± 0.26 to 4.76 ± 0.24. In the dapagliflozin group, the respective decreases were from 4.33 ± 1.59 to 3.62 ± 1.14 for CRI-I, from 2.90 ± 1.32 to 2.27 ± 0.96 for CRI-II, from 3.33 ± 1.59 to 2.62 ± 1.14 for AC, and from 4.79 ± 0.29 to 4.74 ± 0.25 for TyG. All of these changes were statistically significant at *p* < 0.001. By week 12, there were no significant differences between the two drugs in terms of these indices (*p* > 0.05) (see Table 2). Changes in atherogenic indices from baseline to week 12 are also shown in Figure 3.

When the rates of change relative to baseline were evaluated (see Table 3), the percentage changes in lipid parameters and derived indices were very similar between empagliflozin- and dapagliflozin-treated patients, with no significant differences between groups (all *p* > 0.05).

Fasting plasma glucose levels significantly decreased from baseline to week 12 in both empagliflozin (*p* = 0.004) and dapagliflozin (*p* < 0.001) groups, with no significant difference between groups (*p* = 0.480) (glucose values were also analyzed as a component of the TyG index calculation).

Since baseline HDL-C, BUN, creatinine, and uric acid levels differed significantly between groups, we performed an ANCOVA to adjust for these covariates. After adjustment, there was no significant between-group difference in week 12 HDL-C (adjusted means: 48.4 vs. 48.5 mg/dL; mean difference −0.11 [95% CI −1.94 to 1.72], *p* = 0.904). Baseline HDL-C was a strong predictor of week 12 HDL-C (*p* < 0.001), whereas BUN, creatinine, and uric acid were not significant covariates, except for a modest effect of uric acid (*p* = 0.037, partial η^2^ = 0.011).

### 3.4. Changes in Lipid Risk Categories

At 12 weeks, when the patient distribution across risk categories (optimal, borderline-high, and high-risk ranges) was compared with baseline, both SGLT2 inhibitors shifted a significant proportion of patients to lower risk categories (see Table 4):

- TC: In the empagliflozin group, the proportion of patients with TC below 200 mg/dL increased from 58.7% to 77.6% (*p* < 0.001). Those with levels between 200 and 239 mg/dL decreased from 24.4% to 14.9% (*p* = 0.008), and those with levels above 240 mg/dL decreased from 16.9% to 7.5% (*p* < 0.01). In the dapagliflozin group, the proportion of patients with TC < 200 mg/dL increased from 63.3% to 85.5%, while those with 200–239 mg/dL declined from 23% to 8.5% and those with ≥240 mg/dL from 13.8% to 6% (all *p* < 0.001).
- LDL-C: In the empagliflozin group, the proportion of patients with LDL-C < 100 mg/dL increased from 25.9% to 45.8% (*p* < 0.001); the proportion with 130–159 mg/dL decreased from 21.9% to 12.9% (*p* = 0.013); and the proportion with ≥160 mg/dL decreased from 24.4% to 7% (*p* < 0.001). In the dapagliflozin group, the proportion of patients with LDL-C < 100 mg/dL increased from 35% to 61.5% (*p* < 0.001), while the proportion with 130–159 mg/dL decreased from 16.6% to 10.6% (*p* = 0.027) and the proportion with ≥160 mg/dL decreased from 22.6% to 7.4% (*p* < 0.001).
- HDL-C: In the empagliflozin group, HDL-C distribution did not change significantly: patients with >60 mg/dL remained at 20.4%; those with 40–59 mg/dL rose from 51.2% to 55.2%; and those with <40 mg/dL fell from 28.4% to 24.4% (all *p* > 0.05). In the dapagliflozin group, the proportion of patients with HDL-C < 40 mg/dL decreased from 37.1% to 28.3% (*p* < 0.001), while the proportion with 40–59 mg/dL increased from 45.9% to 54.8% (*p* = 0.004). The proportion with >60 mg/dL remained unchanged at 17% (*p* = 1.000).
- TG: In the empagliflozin group, the proportion of patients with TG < 150 mg/dL increased from 63.7% to 71.1%, while those with 150–499 mg/dL decreased from 36.3% to 28.9% (*p* = 0.029). In the dapagliflozin group, the proportion of patients with TG < 150 mg/dL increased from 66.4% to 73.5%, while the proportion with 150–499 mg/dL decreased from 33.6% to 26.5% (*p* = 0.009). No patients in either group had TG > 500 mg/dL.

### 3.5. Changes in Risk Categories of Atherogenic Indices

At week 12, changes in patient distribution according to thresholds of lipid-derived formulas were as follows (see Table 5):

- AIP: Considering the low (below 0.11), intermediate (between 0.11 and 0.21), and high (above 0.21) risk categories, the proportion of patients in the empagliflozin group who remained in the high-risk category decreased only from 85.1% to 82.6%, which was not statistically significant (*p* = 0.369). The proportion in the low-risk category increased from 7.5% to 10% (*p* = 0.197), while the proportion in the intermediate-risk category remained at 7.5%. In the dapagliflozin group, the proportion in the intermediate-risk category decreased significantly from 8.8% to 4.2% (*p* = 0.005), while there was a borderline, non-significant increase in the high-risk category (from 80.9% to 84.5%, *p* = 0.059), and a slight, non-significant increase in the low-risk category (from 10.2% to 11.3%, *p* = 0.532).
- CRI-I: In the empagliflozin group, patients in the low (<3.5) risk category increased from 31.3% to 53.2% and those in the high (>3.5) risk category decreased from 68.7% to 46.8% (both *p* < 0.001). In the dapagliflozin group, the proportion of patients in the low-risk category increased from 35.3% to 57.2%, while the proportion in the high-risk category decreased from 64.7% to 42.8% (both *p* < 0.001).
- CRI-II: A significant shift occurred in both the empagliflozin and dapagliflozin groups from the high (>3.0) risk category to the low (<3.0) category. In the empagliflozin group specifically, the proportion of low-risk patients increased from 61.7% to 82.6%, while the proportion of high-risk patients decreased from 38.3% to 17.4% (*p* < 0.001). In the dapagliflozin group, the proportion of low-risk patients increased from 58% to 78.4%, while the proportion of high-risk patients decreased from 42% to 21.6% (*p* < 0.001).
- AC: In the empagliflozin group, the proportion of patients in the low (<3.0) risk category increased from 49.3% to 70.6%, while the proportion in the high (>3.0) risk category decreased from 50.7% to 29.4% (*p* < 0.001). In the dapagliflozin group, low-risk patients increased from 47% to 71.7% and high-risk patients decreased from 53% to 28.3% (*p* < 0.001).
- TyG index: No statistically significant changes were observed in either group (*p* > 0.05). In the empagliflozin group, the proportion of patients in the low (<4.5) risk category decreased from 10% to 9.5%, while the proportion in the high (>4.5) risk category increased from 90% to 90.5% (*p* = 0.847). In the dapagliflozin group, the proportion in the low-risk category decreased from 16.6% to 14.1%, while the proportion in the high-risk category increased from 83.4% to 85.9% (*p* = 0.250).

## 4. Discussion

The potential role of SGLT2 inhibitors in the prevention and treatment of atherosclerosis has been attracting increasing attention. However, heterogeneous findings have been reported regarding their effects on lipid profiles. While most studies indicate favorable effects on lipid metabolism, some have reported unexpected increases in LDL-C and variability in other lipid parameters, leading to ongoing debate [18,19].

Findings have varied across prior studies, with some reporting decreases and others reporting increases in lipid profiles. For dapagliflozin, results have been heterogeneous: a single-center retrospective study reported reductions in TC, LDL-C, and TG at 3- and 6-month follow-up [35], whereas a 24-week trial with 4401 patients showed increases in TC, LDL-C, and HDL-C with a decrease in TG [36]. Similarly, a meta-analysis by Bechmann et al. and a 24-week randomized clinical trial reported increases in TC, LDL-C, and HDL-C [37,38], while a retrospective comparative study also demonstrated rises in both HDL-C and LDL-C [39]. By contrast, in a 12-week prospective study, Hayashi et al. observed no significant change in LDL-C but an increase in HDL-C [40]. Comparable heterogeneity has been observed with empagliflozin: a randomized controlled trial with 7028 patients reported increases in LDL-C and HDL-C alongside TG reduction [8], a pooled analysis of ten RCTs showed a 4.5–6.5% rise in LDL-C [41], and other short-term studies have also reported heterogeneous effects, with modest increases in LDL-C and variable changes in TC, HDL-C, and TG [42].

In light of this heterogeneous literature, we evaluated and compared the effects of empagliflozin and dapagliflozin on lipid profiles and atherogenic indices in patients with heart failure with preserved ejection fraction and a history of CABG. At baseline, the groups were balanced in terms of demographic and clinical characteristics, except for a significantly lower HDL-C level in the dapagliflozin group. Both drugs significantly reduced TC and LDL-C after 12 weeks, while no significant changes were observed in HDL-C or TG.

At week 12, the mean TC level was significantly lower in the dapagliflozin group, suggesting a relatively more favorable cardiovascular risk profile compared with empagliflozin. Although baseline HDL-C was significantly higher in the dapagliflozin group, both groups showed only slight, non-significant increases over time. Importantly, after adjusting for baseline HDL-C, BUN, creatinine, and uric acid, week 12 HDL-C outcomes did not differ significantly between the groups, confirming that baseline imbalances did not bias the overall results. Consistent with this, BUN and creatinine were not significant predictors, and uric acid showed only a modest effect without altering the overall findings.

When analyzed by threshold-based risk categories, both drugs shifted TC and LDL-C levels of most patients into the optimal range. Dapagliflozin provided a more pronounced proportional improvement in raising HDL-C into the normal range compared with empagliflozin (6% vs. 2.8%); however, absolute and percentage changes between groups were not statistically significant. With regard to TG, both agents improved triglyceride levels, enabling a greater proportion of patients to move into the optimal-risk category.

With respect to atherogenic indices, both SGLT2 inhibitors significantly improved CRI-I, CRI-II, AC, and the TyG index, whereas no significant change was observed in AIP. There were no significant differences between empagliflozin and dapagliflozin in any of these parameters. These findings suggest that both agents share a similar effect profile on lipid-related risk markers and that the observed improvements are more likely attributable to a class effect rather than drug-specific actions.

The lack of significant change in AIP appears consistent with the absence of statistically significant alterations in TG and HDL-C levels. In contrast, the improvement observed in the TyG index may primarily reflect glycemic effects, as this parameter incorporates fasting glucose in addition to triglycerides [43]. In support of this, fasting plasma glucose levels significantly decreased from baseline to week 12 in both treatment groups (empagliflozin, *p* = 0.004; dapagliflozin, *p* < 0.001), with no significant between-group difference (*p* = 0.480), providing a plausible explanation for the within-group improvement observed in TyG. The relatively narrow variance of the TyG index compared with the markedly wide variance of AIP may be related to the mathematical structure of these indices [44,45]. In particular, the logarithmic formula underlying AIP makes it sensitive to low HDL-C values, which may disproportionately amplify the index and account for this variability [25,44].

In terms of risk categories, both drugs reduced the proportion of patients in the high-risk categories of CRI-I, CRI-II, and AC, while increasing those in the low-risk categories. For AIP, patients in both groups largely remained in the high-risk category. Although absolute reductions in the TyG index were observed, there was no increase in the number of patients moving into the low-risk category. Overall, since lipid-derived indices are considered more reliable predictors of cardiovascular risk than classical lipid parameters, the improvement observed with both drugs in a population entirely on statin therapy suggests that they may provide an additional benefit in reducing residual cardiovascular risk beyond statin therapy.

Because all patients in our study were receiving statin therapy, the independent effects of empagliflozin and dapagliflozin on lipid metabolism could not be isolated. Nevertheless, the consistent improvements observed in CRI-I, CRI-II, AC, and TyG despite background statin use may suggest a possible synergistic effect. Future studies including non-statin users or populations treated with varying statin intensities would help clarify this relationship.

To our knowledge, this is the first study in the literature to compare the effects of empagliflozin and dapagliflozin on atherogenic indices in patients with heart failure and a history of CABG. The most notable finding of our study is that both drugs significantly improved key lipid-based indices, with largely comparable outcomes, although a modestly greater reduction in TC was observed with dapagliflozin (*p* = 0.044). This finding suggests that the cardioprotective effects of SGLT2 inhibitors may not be limited to mechanisms such as glycemic control, blood pressure lowering, and diuresis [11,13,14,17], but may also be mediated through favorable effects on lipid metabolism and atherogenic indices. In addition, the pleiotropic actions of SGLT2 inhibitors—including anti-inflammatory, antioxidative, endothelial-protective, and anti-arrhythmic effects—have been highlighted in recent reviews [46], providing a mechanistic explanation for their consistent cardiovascular benefits observed in major outcome trials such as EMPA-REG [8], DAPA-HF [47], and DELIVER [48].

The EMPA-REG OUTCOME trial reported modest increases in LDL-C and HDL-C with empagliflozin, whereas DAPA-HF and DELIVER demonstrated largely neutral lipid effects. Notably, our cohort—composed entirely of patients on moderate-to-high intensity statins—showed significant reductions in TC and LDL-C, with no meaningful changes in HDL-C or TG. This finding contrasts with the LDL-C increase observed in EMPA-REG. The reason for this discrepancy cannot be clearly explained with the available data; factors such as patient population, study design, methodological differences, the universal use of moderate-to-high intensity statin therapy, potential effects on LDL particle size or density, unidentified pharmacological interactions, or other yet unknown variables may have influenced the results. Future studies evaluating LDL subfractions may help clarify this paradox.

The main limitations of our study are its retrospective design, single-center nature, and relatively short follow-up period. In the long term, the clinical significance of the effects of SGLT2 inhibitors on lipid profiles may change over time. Metabolic adaptation processes, concomitant interventions such as diet, exercise, and other medications, as well as individual patient factors, may play an important role. Furthermore, since all patients were receiving statin therapy, the independent effects of empagliflozin and dapagliflozin on lipid metabolism could not be clearly distinguished. Moreover, only standard doses (10 mg/day) were evaluated in this study; potential dose–response relationships remain to be clarified. Given that our findings are derived exclusively from a CABG cohort with preserved ejection fraction, caution should be exercised in extrapolating these results to patients with HFrEF or without prior CABG. In addition, the long-term effects of these agents on cardiovascular outcomes were not evaluated in our study and should be addressed in larger, prospective studies.

Despite these limitations, our study contributes to the literature by demonstrating, for the first time, the comparative effects of empagliflozin and dapagliflozin on lipid profiles and atherogenic indices in patients with heart failure and a history of CABG.

In conclusion, whether the lipid-related changes observed in our study will translate into long-term clinically meaningful reductions in cardiovascular risk remains uncertain. Nevertheless, the consistent improvements across multiple atherogenic indices suggest that SGLT2 inhibitors may have the potential to provide additional cardioprotective benefits beyond glycemic and hemodynamic mechanisms.

## 5. Conclusions

In this study, conducted in patients with heart failure and a history of CABG, the effects of empagliflozin and dapagliflozin on lipid profiles and atherogenic indices were compared. Both agents significantly reduced TC and LDL-C levels after 12 weeks of treatment. Although no statistically significant changes were observed in HDL-C and TG, both drugs provided substantial improvements in CRI-I, CRI-II, AC, and the TyG index. In contrast, no significant change was observed in AIP. Beyond absolute improvements, both agents also contributed to favorable shifts in the risk categories of lipid-derived indices. Dapagliflozin showed a greater reduction in TC compared with empagliflozin (*p* = 0.044). Overall, these findings suggest that both empagliflozin and dapagliflozin exert favorable effects on lipid parameters and atherogenic indices, with largely comparable outcomes. To our knowledge, this is the first study in the literature to evaluate and compare the effects of empagliflozin and dapagliflozin on lipid-derived indices in this specific patient group.

These findings may have implications for clinical practice, suggesting that the choice between empagliflozin and dapagliflozin may be guided by factors other than lipid modulation, given their comparable effects on lipid-derived indices. However, the generalizability of these results to non-CABG or HFrEF populations remains uncertain and requires further validation. Confirmation of our findings and a better understanding of the long-term effects of these agents on lipid metabolism and cardiovascular risk will require larger-scale, multicenter, prospective studies.

## Figures and Tables

**Table 1:** Baseline demographic, clinical, medical treatment, and laboratory characteristics of patients treated with empagliflozin or dapagliflozin.

Variables	Empagliflozin (*n* = 201)	Dapagliflozin (*n* = 283)	Total (*n* = 484)	*p* Value	95% CI Phi Value
Age, years	61.41 ± 10.58	63.08 ± 11.24	62.97 ± 11.04	0.151	−0.31–3.66
≥60 years, *n* (%)	117 (58.2)	168 (59.4)	285 (59.0)	0.799	0.001
Sex, *n* (%)					
Male	120 (59.7)	186 (65.7)	306 (63.2)	0.176	0.062
Female	81 (40.3)	97 (34.3)	178 (36.8)		
Comorbidities, *n* (%)					
Hypertension (HT)	133 (66.2)	178 (62.9)	311 (64.3)	0.459	0.034
Diabetes mellitus (DM)	125 (62.2)	181 (64.0)	306 (63.2)	0.691	−0.018
Hyperlipidemia (HL)	122 (60.7)	244 (86.4)	366 (84.5)	0.216	−0.056
Smoking	54 (26.9)	55 (19.4)	89 (19.0)	0.481	−0.032
Atrial fibrillation (AF)	41 (20.4)	71 (25.1)	112 (23.1)	0.228	−0.055
Medications, *n* (%)					
Beta-blocker	162 (80.6)	217 (76.7)	379 (78.3)	0.303	−0.050
Aldactone (MRA)	80 (39.8)	110 (38.7)	190 (39.3)	0.812	−0.011
Furosemide	127 (63.2)	187 (65.8)	314 (64.9)	0.546	0.027
Torasemide	46 (22.9)	66 (23.2)	112 (23.2)	0.927	0.004
Calcium channel blocker	45 (22.4)	68 (23.9)	113 (23.3)	0.690	0.018
ACE-i/ARB	165 (82.1)	231 (81.3)	396 (81.9)	0.833	−0.010
Acetylsalicylic acid	201 (100)	283 (100)	484 (100)	1.000	-
Statin therapy				0.409	−0.38
High dose statin therapy	161 (80.1)	235 (83)	396 (81.8)		
Moderate dose statin therapy	40 (19.9)	48 (17)	88 (18.2)		
Laboratory data					
Fasting plasma glucose (mg/dL)	122.23 ± 44.04	122.68 ± 44.28	122.49 ± 44.13	0.776	−7.55–8.46
HbA1c, %	6.64 ± 1.37	6.65 ± 1.29	6.65 ± 1.32	0.656	−0.23–0.25
BUN (mg/dL)	17.25 ± 6.95	18.72 ± 8.29	18.11 ± 7.78	0.030	0.09–2.84
Creatinine (mg/dL)	0.89 ± 0.22	0.97 ± 0.22	0.94 ± 0.23	0.001	0.04–0.12
Uric acid (mg/dL)	5.72 ± 1.55	6.35 ± 1.79	6.10 ± 1.71	0.001	0.26–0.94
Lipids					
Total cholesterol (TC), mg/dL	193.81 ± 50.25	186.19 ± 49.59	189.35 ± 49.95	0.087	−16.65–1.41
LDL-C, mg/dL	130.59 ± 45.61	123.27 ± 44.48	126.31 ± 45.05	0.061	−15.46–0.82
HDL-C, mg/dL	48.57 ± 13.56	46.38 ± 15.42	47.29 ± 14.70	0.018	−4.84–0.47
Triglycerides (TG), mg/dL	145.31 ± 66.51	139.53 ± 72.86	141.93 ± 70.28	0.127	18.51–6.96
AIP	0.45 ± 0.25	0.45 ± 0.28	0.45 ± 0.27	0.897	−0.05–0.45
CRI-I	4.21 ± 1.33	4.33 ± 1.59	4.28 ± 1.49	0.690	−0.13–0.38
CRI-II	2.85 ± 1.16	2.90 ± 1.32	2.88 ± 1.09	0.948	−0.17–0.27
AC	3.21 ± 1.33	3.33 ± 1.59	3.28 ± 1.49	0.690	−0.13–0.38
TyG index	4.82 ± 0.26	4.79 ± 0.29	4.80 ± 0.28	0.223	−0.08–0.02

Values are presented as mean ± standard deviation (SD) or number (percentage). *p* Values indicate between-group comparisons. For continuous variables, 95% confidence intervals are provided; for categorical variables, phi coefficients are reported. Abbreviations: HT, hypertension; DM, diabetes mellitus; HL, hyperlipidaemia; AF, atrial fibrillation; MRA, mineralocorticoid receptor antagonist; ACE-i, angiotensin-converting enzyme inhibitor; ARB, angiotensin receptor blocker; FPG, fasting plasma glucose; HbA1c, glycated haemoglobin; BUN, blood urea nitrogen; TC, total cholesterol; LDL-C, low-density lipoprotein cholesterol; HDL-C, high-density lipoprotein cholesterol; TG, triglycerides; AIP, atherogenic index of plasma; CRI-I, Castelli Risk Index I; CRI-II, Castelli Risk Index II; AC, atherogenic coefficient; TyG, triglyceride-glucose index.

**Table 2:** Changes in lipid profiles and atherogenic indices from baseline to week 12 in patients treated with empagliflozin or dapagliflozin.

Variables	Empagliflozin (*n* = 201)	Dapagliflozin (*n* = 283)	*p* Value (Between Groups)	95% CI
TC, mg/dL				
Week 0 (Baseline)	193.81 ± 50.25	186.19 ± 49.59	0.087	−16.65–1.41
Week 12	169.66 ± 44.55	162.31 ± 38.31	0.044	−14.78–0.08
*p* (within-group)	<0.001	<0.001		
95% CI	17.37–30.92	18.41–29.33		
LDL-C, mg/dL				
Week 0 (Baseline)	130.59 ± 45.61	123.27 ± 44.48	0.061	−15.46–0.82
Week 12	105.15 ± 38.74	100.38 ± 34.20	0.132	−11.31–1.78
*p* (within-group)	<0.001	<0.001		
95% CI	19.00–31.87	17.48–28.29		
HDL-C, mg/dL				
Week 0 (Baseline)	48.57 ± 13.56	46.38 ± 15.42	0.018	−4.84–0.47
Week 12	49.28 ± 13.84	47.25 ± 12.97	0.150	−4.45–0.38
*p* (within-group)	>0.05	>0.05		
95% CI	−1.81–0.39	−2.23–0.51		
TG, mg/dL				
Week 0 (Baseline)	145.31 ± 66.51	139.53 ± 72.86	0.127	18.51–6.96
Week 12	131.90 ± 54.94	128.34 ± 56.43	0.392	−6.33–8.48
*p* (within-group)	>0.05	>0.05		
95% CI	4.18–22.64	5.05–17.34		
AIP				
Week 0 (Baseline)	0.45 ± 0.25	0.45 ± 0.28	0.150	−0.05–0.45
Week 12	0.41 ± 0.24	0.41 ± 0.24	0.987	−0.04–0.05
*p* (within-group)	>0.05	>0.05		
95% CI	0.01–0.07	0.01–0.06		
CRI-I				
Week 0 (Baseline)	4.21 ± 1.33	4.33 ± 1.59	0.690	−0.13–0.38
Week 12	3.60 ± 1.01	3.62 ± 1.14	0.712	−0.17–0.22
*p* (within-group)	<0.001	<0.001		
95% CI	0.44–0.77	0.55–0.87		
CRI-II				
Week 0 (Baseline)	2.85 ± 1.16	2.90 ± 1.32	0.948	−0.17–0.27
Week 12	2.24 ± 0.90	2.27 ± 0.96	0.826	−0.14–0.18
*p* (within-group)	<0.001	<0.001		
95% CI	0.45–0.75	0.48–0.77		
AC				
Week 0 (Baseline)	3.21 ± 1.33	3.33 ± 1.59	0.690	−0.13–0.38
Week 12	2.60 ± 1.01	2.62 ± 1.14	0.712	−0.17–0.22
*p* (within-group)	<0.001	<0.001		
95% CI	0.44–0.77	0.54–0.87		
TyG index				
Week 0 (Baseline)	4.82 ± 0.26	4.79 ± 0.29	0.241	−0.08–0.02
Week 12	4.76 ± 0.24	4.74 ± 0.25	0.335	−0.06–0.02
*p* (within-group)	<0.001	<0.001		
95% CI	0.03–0.10	0.03–0.08		
Fasting plasma glucose, mg/dL				
Week 0 (Baseline)	122.23 ± 44.04	122.68 ± 44.28	0.776	−7.55–8.46
Week 12	114.65 ± 34.13	112.83 ± 28.78	0.480	−7.46–3.81
*p* (within-group)	0.004	<0.001		
95% CI	2.47–12.68	5.73–13.96		

Values are presented as mean ± standard deviation (SD). *p* Values indicate comparisons within groups (baseline vs. week 12) and between groups (empagliflozin vs. dapagliflozin). For each parameter, 95% confidence intervals (CI) are provided. Abbreviations: TC, total cholesterol; LDL-C, low-density lipoprotein cholesterol; HDL-C, high-density lipoprotein cholesterol; TG, triglycerides; AIP, atherogenic index of plasma; CRI-I, Castelli Risk Index I; CRI-II, Castelli Risk Index II; AC, atherogenic coefficient; TyG, triglyceride-glucose index.

**Table 3:** Percentage changes from baseline to week 12 in lipid profiles and atherogenic indices in patients treated with empagliflozin or dapagliflozin.

Variables	Change from Baseline (%) in Lipid Parameters (mg/dL) and Atherogenic Indices		*p* Value
	Empagliflozin (*n* = 201)	Dapagliflozin (*n* = 283)	
TC, mg/dL	−9.40 ± 23.99 (95% CI, −12.74 to −6.06)	−9.25 ± 22.00 (95% CI, −11.82 to −6.67)	0.358
LDL-C, mg/dL	−13.57 ± 33.54 (95% CI, −18.23 to −8.91)	−11.08 ± 34.03 (95% CI, −15.07 to −7.09)	0.163
HDL-C, mg/dL	2.84 ± 19.24 (95% CI, 0.16 to 5.52)	6.06 ± 26.83 (95% CI, 2.92 to 9.20)	0.401
TG, mg/dL	0.17 ± 44.16 (95% CI, −5.97 to 6.31)	1.24 ± 38.40 (95% CI, −3.25 to 5.74)	0.307
AIP	20.79 ± 360.64 (95% CI, −29.37 to 70.95) −4.66 [−28.00–17.60] *	26.67 ± 237.29 (95% CI, −1.10 to 54.43) −4.73 [−27.35–18.43] *	0.750
CRI-I	−9.90 ± 26.35 (95% CI, −13.57 to −6.24)	−10.70 ± 26.88 (95% CI, −13.84 to −7.55)	0.394
CRI-II	−13.83 ± 36.08 (95% CI, −18.85 to −8.82)	−10.67 ± 52.19 (95% CI, −16.77 to −4.56)	0.225
AC	−9.56 ± 50.43 (95% CI, −16.57 to −2.54)	−10.82 ± 45.55 (95% CI, −16.15 to −5.49)	0.357
TyG index	−1.24 ± 4.96 (95% CI, −1.93 to −0.55)	−1.02 ± 4.48 (95% CI, −1.54 to −0.50)	0.592

Values are presented as mean ± standard deviation (SD). * For AIP, due to the non-normal distribution, data are also expressed as median [interquartile range, Q1–Q3]. *p* Values indicate comparisons between groups. Abbreviations: TC, total cholesterol; LDL-C, low-density lipoprotein cholesterol; HDL-C, high-density lipoprotein cholesterol; TG, triglycerides; AIP, atherogenic index of plasma; CRI-I, Castelli Risk Index I; CRI-II, Castelli Risk Index II; AC, atherogenic coefficient; TyG, triglyceride-glucose index.

**Table 4:** Distribution of patients across risk categories of lipid parameters from baseline to week 12 in the empagliflozin and dapagliflozin groups.

Variables	Categories	Empagliflozin (*n* = 201)	*p* Value	Dapagliflozin (*n* = 283)	*p* Value
TC, mg/dL, *n* (%)	<200 mg/dL	118 (58.7) to 156 (77.6)	<0.001	179 (63.3) to 242 (85.5)	<0.001
	200–239 mg/dL	49 (24.4) to 30 (14.9)	0.008	65 (23) to 24 (8.5)	<0.001
	>240 mg/dL	34 (16.9) to 15 (7.5)	<0.001	39 (13.8) to 17 (6)	<0.001
LDL-C, mg/dL, *n * (%)	<100 mg/dL	52 (25.9) to 92 (45.8)	<0.001	99 (35) to 174 (61.5)	<0.001
	130–159 mg/dL	44 (21.9) to 26 (12.9)	0.013	47 (16.6) to 30 (10.6)	0.027
	>160 mg/dL	49 (24.4) to 14 (7)	<0.001	64 (22.6) to 21 (7.4)	<0.001
HDL-C, mg/dL, *n* (%)	>60 mg/dL	41 (20.4) to 41 (20.4)	1.000	48 (17) to 48 (17)	1.000
	40–59 mg/dL	103 (51.2) to 111 (55.2)	0.248	130 (45.9) to 155 (54.8)	0.004
	<40	57 (28.4) to 49 (24.4)	0.157	105 (37.1) to 80 (28.3)	<0.001
TG, mg/dL, *n* (%)	<150 mg/dL	128 (63.7) to 143 (71.1)	0.029	188 (66.4) to 208 (73.5)	0.009
	150–499 mg/dL	73 (36.3) to 58 (28.9)	0.029	95 (33.6) to 75 (26.5)	0.009
	>500 mg/dL	-	-	-	-

Values are expressed as number (percentage) of patients within each category. Arrows indicate changes from baseline to week 12. *p*-values represent within-group comparisons of category shifts. Abbreviations: TC, total cholesterol; LDL-C, low-density lipoprotein cholesterol; HDL-C, high-density lipoprotein cholesterol; TG, triglycerides.

**Table 5:** Distribution of patients across risk categories of atherogenic indices from baseline to week 12 in empagliflozin and dapagliflozin groups.

Variables	Categories	Empagliflozin (*n* = 201)	*p* Value	Dapagliflozin (*n* = 283)	*p* Value
AIP, *n* (%)	<0.11	15 (7.5) to 20 (10)	0.197	29 (10.2) to 32 (11.3)	0.532
	0.11–0.21	15 (7.5) to 15 (7.5)	1.000	25 (8.8) to 12 (4.2)	0.005
	>0.21	171 (85.1) to 166 (82.6)	0.369	229 (80.9)) to 239 (84.5)	0.059
CRI-I, *n* (%)	<3.5	63 (31.3) to 107 (53.2)	<0.001	100 (35.3) to 162 (57.2)	<0.001
	>3.5	138 (68.7) to 94 (46.8)	<0.001	183 (64.7) to 121 (42.8)	<0.001
CRI-II, *n* (%)	<3.0	124 (61.7) to 166 (82.6)	<0.001	164 (58) to 222 (78.4)	<0.001
	>3.0	77 (38.3) to 35 (17.4)	<0.001	119 (42) to 61 (21.6)	<0.001
AC, *n* (%)	<3.0	99 (49.3) to 142 (70.6)	<0.001	133 (47) to 203 (71.7)	<0.001
	>3.0	102 (50.7) to 59 (29.4)	<0.001	150 (53) to 80 (28.3)	<0.001
TyG Index, *n* (%)	<4.5	20 (10) to 19 (9.5)	0.847	47 (16.6) to 40 (14.1)	0.250
	>4.5	181 (90) to 182 (90.5)	0.847	236 (83.4) to 243 (85.9)	0.250

Values are expressed as number (percentage) of patients within each risk category. Arrows indicate changes from baseline to week 12. *p* Values represent within-group comparisons of category shifts. Abbreviations: AIP, atherogenic index of plasma; CRI-I, Castelli Risk Index I; CRI-II, Castelli Risk Index II; AC, atherogenic coefficient; TyG, triglyceride-glucose index.

## Data Availability

The data presented in this study are available on reasonable request from the corresponding author. The data are not publicly available due to privacy and ethical restrictions.
